# Muslim Communities Learning About Second-hand Smoke in Bangladesh (MCLASS II): a combined evidence and theory-based plus partnership intervention development approach

**DOI:** 10.1186/s40814-022-01100-5

**Published:** 2022-07-02

**Authors:** Ian Kellar, Zunayed Al Azdi, Cath Jackson, Rumana Huque, Noreen Dadirai Mdege, Kamran Siddiqi, on behalf of the MCLASS II Trial Team

**Affiliations:** 1 School of Psychology, Faculty of Medicine & Health, University of Leeds, Leeds, LS2 9JT UK; 2 ARK Foundation, Suite C-3, C-4, House number 06, Road 109, Dhaka, 1212 Bangladesh; 3 Valid Research Ltd., Sandown House, Sandbeck Way, Wetherby, LS22 7DN UK; 4 Department of Health Sciences, Faculty of Sciences, University of York, York, YO10 5DD UK; 5 Department of Economics, Dhaka University, Dhaka, Bangladesh; 6 Hull York Medical School, University of York, Heslington, York, YO10 5DD UK

**Keywords:** Smoke-free home, Mosque, Intervention development

## Abstract

**Introduction:**

Deaths from second-hand smoke (SHS) exposure are increasing, but there is not sufficient evidence to recommend a particular SHS intervention or intervention development approach. Despite the available guidance on intervention reporting, and on the role and nature of pilot and feasibility studies, partial reporting of SHS interventions is common. The decision-making whilst developing such interventions is often under-reported. This paper describes the processes and decisions employed during transitioning from the aim of adapting an existing mosque-based intervention focused on public health messages, to the development of the content of novel community-based Smoke-Free Home (SFH) intervention. The intervention aims to promote smoke-free homes to reduce non-smokers’ exposure to SHS in the home via faith-based messages.

**Methods:**

The development of the SFH intervention had four sequential phases: in-depth interviews with adults in households in Dhaka, identification of an intervention programme theory and content with Islamic scholars from the Bangladesh Islamic Foundation (BIF), user testing of candidate intervention content with adults, and iterative intervention development workshops with Imams and khatibs who trained at the BIF.

**Results:**

It was judged inappropriately to take an intervention adaptation approach. Following the identification of an intervention programme theory and collaborating with stakeholders in an iterative and collaborative process to identify barriers, six potentially modifiable constructs were identified. These were targeted with a series of behaviour change techniques operationalised as Quranic verses with associated health messages to be used as the basis for Khutbahs. Following iterative user testing, acceptable intervention content was generated.

**Conclusion:**

The potential of this community-based intervention to reduce SHS exposure at home and improve lung health among non-smokers in Bangladesh is the result of an iterative and collaborative process. It is the result of the integration of behaviour change evidence and theory and community stakeholder contributions to the production of the intervention content. This novel combination of intervention development frameworks demonstrates a flexible approach that could provide insights for intervention development in related contexts.

**Supplementary Information:**

The online version contains supplementary material available at 10.1186/s40814-022-01100-5.

## Introduction

Historically, behaviour change intervention content is under-reported [1], impacting replicability, subsequent development, and scalability. A recent review of second-hand smoke (SHS) intervention studies [2] indicated that partial reporting of SHS interventions is common. It was recommended that intervention reporting guidelines are adhered to and that comprehensive reporting of behaviour change techniques (BCTs) and the provision of a logic model linking BCTs to the intervention theory of change is mandated. The need to be pragmatic in resource-limited contexts is common in intervention development [3]. The decisions taken in these contexts and elsewhere may enlighten those seeking to understand what leads to successful intervention development. A range of theoretical models and intervention development approaches to protect children from SHS [4] have been proposed, but recent reviews of smoke-free homes (SFH) [5, 6] and of SHS interventions for children [7] have not provided the basis for specific recommendations. Hoddinott [8] suggests that a greater understanding of the effectiveness of interventions will result from transparent reporting of how stakeholder groups are involved in decision-making during the development of complex interventions. This paper describes the process of developing the content of a novel mosque-based smoke-free home (SFH) intervention in Bangladesh that has subsequently been trialled [9].

### Key messages regarding feasibility

1) Previous work had identified concerns around the feasibility of developing smoke-free homes messages that could be delivered in mosques.

2) Our approach demonstrates it is feasible to develop explicitly faith-based messages for use in mosques by working iteratively with stakeholder groups from religious communities.

3) The reported intervention development utilised a 4-phase process for working with stakeholders from religious communities to develop faith-based intervention content.

## Background

SHS is the combination of emissions of smoke emitted between a puff of lit tobacco and the smoke that is exhaled by smokers [10]. Children’s risks from asthma [11], acquiring lower respiratory tract infections [12, 13], and tuberculosis [14, 15] are all increased by exposure to SHS. Children living in smoking households are also at high risk of becoming adult smokers later [16]. Childhood exposure to SHS is strongly associated with the prevalence of adult smoking [17].

Whilst between 1990 and 2006, the estimated number of deaths attributed to SHS fell, it has subsequently increased, driven by increases in SHS exposure in South Asia, East Asia, and the Pacific [18]. The WHO estimates that 1.2 million deaths per year are attributable to non-smokers being exposed to SHS [19]. This research focuses on a setting-based approach [20], focussing on engendering a health-supporting environment [21] to protect non-smoking adults and children from the harms of SHS in their homes. There have been calls for research into the efficacy of health interventions that are delivered by Imams or in mosques [22, 23]. The work builds on the findings of a pilot trial conducted in England which concluded that an SFH intervention was acceptable to Muslim communities and feasible to deliver in mosques [24]. In the present work, the intervention development explicitly aimed to result in faith-based material directly targeted at smokers via faith leaders based in mosques (Imams and khatibs) for the planned trial [25] (MRC RGMR/P008941/1).

## Methods

### Development approach

The starting point of the intervention development approach was material arising from the UK-based MCLASS trial [26], for which a package of SFH materials was developed that drew upon consensus around the religious prohibition of the use of tobacco products among Muslims [27, 28], and evidence that a complex intervention that included a mosque-based component had promising effects on SFH prevalence [29]. The MCLASS intervention took a settings-based approach, seeking to support health-promoting environments. The intervention was tailored to the cultural values of the target population: South-Asian men ill-served by smoking cessation services that do not address cultural sensitivities [30–32]. Relatively few faith setting-based interventions have been developed for mosques [33].

A recent UK Medical Research Council (MRC)-funded project has produced a taxonomy of intervention development approaches for complex interventions [34]. This specified eight categories: partnership, target population-centred, evidence and theory-based, implementation-based, efficiency-based, stepped or phased-based intervention specific, and combination. Our development work does not fit neatly into this taxonomy, in that we had previously undertaken SHS intervention development in the UK [26]. We initially expected to undertake an intervention adaptation approach using the Programme Theory of Adapted Health Interventions [35] making use of the UK-based MCLASS trial materials [26]. However, subsequent process evaluation of the existing intervention [24] raised issues around the acceptability of religious teachers taking on a health promotion role, and it was reported that some participants were unhappy that the mosque was being used as a context for delivering health promotion messages:

When you come to the mosque, you want to pray, you know? And [its’] a place of worship really. And you don’t want to come here and do other things you know? You want to escape from these things you see. (FGD-Men) (p.300)

We subsequently looked to ayah (Quranic verse) for messages that supported SFH so that the messages were drawn from the Quran and would not be jarring for worshippers or out of place in mosques. Given the limited expertise of we in the Quranic scripture, it was felt important to undertake an intervention development process that examined the wider context of smoking and SFH, and following content development, put this before stakeholder groups in Bangladesh for iteration, including those with a scholarly understanding of Quranic scripture.

We elected to undertake a development process that consisted of four phases:

1. Interviews exploring barriers and facilitators of SFH with adults from locations near the planned recruitment sites.
2. Identification of an intervention programme theory and content with Islamic scholars from the Bangladesh Islamic Foundation (BIF) with expertise in Quranic scripture to identify candidate content
3. User testing of candidate intervention content with adults.
4. Iterative intervention development workshops with Imams and khatibs.

### Phase 1—Interviews exploring barriers and facilitators of SFH

Face-to-face interviews were conducted from May to July 2017 in the Mirpur and Gulshan regions of Dhaka city with six men and two women (see Table 1).

**Table 1:** Interview participant characteristics (*n*=8)

Characteristic	Number	%
Sex		
Male	6	75
Female	2	24
Smoking status		
Smoker	6	75
Non-smoker	2	25
Age		
30–39 years	4	50
40–49 years	4	50
Education		
None/primary	4	40
Secondary	2	25
Honours and above	2	25

Drawing upon prior work [36–38] and a relevant systematic review and thematic synthesis [39], a semi-structured interview schedule that explored smoking behaviours, and barriers and facilitators to an SFH intervention delivered within mosques by Imams was developed. Given the aforementioned process evaluation [24] had identified issues around the acceptability and feasibility of the use of mosques to disseminate SHS messages, we took this opportunity to elicit opinions on this. Interviews lasted between 23 and 48 min. They were audio-recorded and fully transcribed then translated from Bangla to English. The interview data were then analysed using deductive content analysis [40]. First a categorisation matrix was developed based on the interview schedule, piloted with one transcript, and set up in Excel. The data were coded to the matrix, and then, each category, e.g., smoking behaviours, was written up.

### Phase 2—Identification of programme theory and content

The basis for the programme theory to guide the development of the content targeting SFH was planned to be selected following the face-to-face interviews. The aim was to identify evidence-based modifiable constructs present within the interview findings and map these to BCTs [41] that seemed likely to result in changes in those constructs based on study team expertise. These BCTs were then operationalised as intervention content with the support of Quranic verses (ayahs) and linked health messages. To seed the programme content design process, we sought advice from a Muslim colleague with knowledge of social cognitive constructs and the BCT taxonomy [41] as to relevant ayahs that supported health messages that could operate as the basis for BCTs. These were fed into the Arabic Quranic Search Tool, which is a semantic search tool for the Quran based on a Quranic ontology [42] to identify a long list of ayahs which matched related concepts. To select from these ayahs and messages, we collaborated with Islamic Scholars from the Bangladesh Islamic Foundation, a government organisation under the Ministry of Religious Affairs in Bangladesh whose role is to spread the values and ideals of Islam among people. The long list of ayahs was screened for those that mapped on to social cognitive constructs within our intervention programme theory. As such, these were ayahs that would support health messages that function as BCTs or prompts to perform BCTs that would potentially result in changes to the intervention programme theory constructs. Subsequently, these ayahs were then expanded upon into statements that could form the suggested basis for a Khutbah (sermon)—the time before Arabic Khutbah during Friday Jumu’ah prayers. The health messages connected ayahs to personal implications for individuals’ faith and tobacco use.

### Phase 3—User testing of candidate intervention content

To test the understanding and acceptability of the selected ayahs and health messages, we employed a user testing methodology [43] using face-to-face interviews. This occurred between September and November 2017 in the Mirpur region of Dhaka. All 12 ayahs and associated health messages were tested with a small sample of men and women (*n*=6, see Table 2) within the communities where we planned to trial the intervention.

**Table 2:** User testing participant characteristics (*n*=6)

Characteristic	Number	%
Sex		
Male	5	83
Female	1	17
Smoking status		
Smoker	3	50
Non-smoker	3	50
Age		
20–29 years	4	66
30–39 years	2	33
Education		
None/primary	2	33
Secondary	2	33
Honours and above	2	33

For each pair of ayah and health messages, the researcher read out the ayah and asked the participant what this meant to them. The health message was subsequently read to them, and questions probing their understanding were asked, including how the message linked to the ayah. Feedback on the clarity of wording and suggestions for improvement were also sought. Interviews lasted between 40 and 70 min. Data analysis was as described in phase 1.

### Phase 4—Iterative intervention development workshops with Imams and khatibs

The iterative workshops were undertaken in two sessions (labelled A and B) with Imams/khatibs from 12 mosques (see Table 3). Imams are those who lead everyday prayers in the mosques. Khatib or khateebs are those who deliver Khutbah and lead the Friday prayers. All of the Imams/khatibs were attendees of the Imam Training Academy, Bangladeshi Islamic Foundation, part of the Ministry of Religious Affairs.

**Table 3:** Imam participant characteristics (*n*-=13)

Characteristic	Number	%
Mosque		
A	6	46
B	7	54
Role in mosque		
Imam	4	31
Mix of roles	9	69
Years of service in mosque		
<10 years	5	38
11–20 years	8	62

We employed the same user-testing methodology applied in Phase 3 [43]. Experience of, and views on, delivering health and behaviour change messages within their religious teaching were also discussed. The two workshops lasted 180 min each. Data analysis was as described in Phase 1.

## Results

### Phase 1—Interviews exploring barriers and facilitators of SFH

#### Smoking behaviours

There was typically one smoker in each participant’s home, often the interview participants themselves. The number of times they smoked in the home ranged from one to eight times a day, usually in the morning and at night, during the day the men were out at work. Some said that they try to smoke on the balcony or in an empty room, which was difficult for the three families who live together in one room. Only one smoker claimed to never smoke in the home.

I felt that the smoke will be harmful for my family members and I stopped smoking inside home. (P01: Male, 35 years, Smoker, highly educated)

#### Barriers and drivers to achieving an SFH

Whilst all interview participants knew of the risks of smoking to the smoker, knowledge of the dangers of SHS varied and was better among the more educated, although they still underestimated the extent of potential harm.

I know that it harms equally others who are around someone who is smoking. That is why I have quit smoking at home totally now. (P01: Male, 35 years, Smoker, highly educated)

The consensus was there were no disadvantages of having an SFH. Participants identified multiple benefits, mentioning particularly the positive impact on the health of family members, especially children. Indeed, this was seen to be the key motivator. Other benefits were seen to be eliminating the smell and improving air quality in the home, reducing the risk of an accidental fire and sons not copying their father’s smoking behaviour.

Everyone loves their children. People would be ready to do anything for the betterment of their children. If they stop smoking at home then the air of that house would not be polluted. Wives and children of smokers will be able to inhale clean air and they will remain healthy. There would not be any bad smell of cigarette smoke in clothing. The overall environment of home will remain very good. (P07: Male, 36 years, Smoker, moderately educated)

The key challenge to achieving an SFH was smokers ignoring requests to smoke outside the home. Several men acknowledged this, whilst one woman spoke of how it would be difficult for women to ask men to smoke outside, suggesting they may not listen or worse, react angrily. She hoped the men would be motivated themselves.

She tells me not to smoke inside home, she has told me. Then, sometimes, I stop smoking inside home, then maybe after a few days, I start smoking in the home again, you know. (P07: Male, 38 years, Smoker, not educated)

Motivating and convincing the smokers would be a challenge, I think. As in our society men are often dominating, it is not likely that all of them will listen, some of them may get angry hearing such things. In some families there might be conflict. If the smokers are motivated enough by themselves, it would be better. (P08: Female, 45 years, Non-smoker, highly educated)

#### Acceptability and feasibility of a mosque-based SHS intervention

All the interview participants thought it was a good idea to educate people about SHS through mosques because of the credibility and influence of the Imam as a religious leader, and the mix of people who would hear the messages. Most had not heard health messages in the mosque before.

Those who have faith in religion go to the mosque, that's why normally they should abide by the rules and regulations of the religion. As the Imam is a religious leader, people listen to him and discuss problems with him, if he talks about smoking, some people will definitely listen to those messages. (P01: Male, 35 years, Smoker, highly educated)

People who go to the mosque regularly and on time are mostly guardians from families, the young generation like us are less in number. So, by them (these guardians) these kinds of messages can spread to others. Another thing would be best if we can make women in our homes more aware and they will definitely be able to make sure that nobody smokes at home. (P06: Male, 34 years, Smoker, moderately educated)

The consensus was that the content of the messages would need to be tailored to the audience. Women and children would need knowledge about SHS to persuade family members not to smoke inside and to protect themselves from smoke, whereas the men would benefit from learning about SHS in the context of Islamic scripture.

Women also need awareness. They will then tell the smoker family members not to smoke inside home. If children get to know the harms of SHS they would then try to protect themselves from second-hand smoking. (P07: Male, 38 years, Smoker, not educated)

The messages should vary. In the mosque the Imam can tell people about these (messages) with hadiths and Quran teachings. But for women there can be other things. For children the message should be in such form that they can communicate with their parents. (P02: Male, 40 years, Smoker, limited ability to read)

In terms of feasibility, the time before Arabic Khutbah (when the largest proportion of a mosque’s congregation attends) was seen as the sensible time to deliver the messages as most men attend then, thus maximising the size of the audience.

We, poor people, rich people, everybody goes to Jum’ah prayer. It's like the Eid day. Old people, younger people, small children gather together. So, it would be good delivering these messages during Jum’ah prayer. Everybody will listen and give importance. (P05: Female, 42 years, Smoker, not educated)

Other ideas for message delivery were Quran classes (for children), Madrasa classes, and other congregations like Milad mahfil (a custom practised by many Muslims as an expression of reverence for Prophet Muhammad (PBUH)) and Waz mahfil (Islamic sermon in the communities) although these were acknowledged to reach fewer people and occur less frequently.

### Phase 2—Identification of programme theory and content

**Fig. 1:**
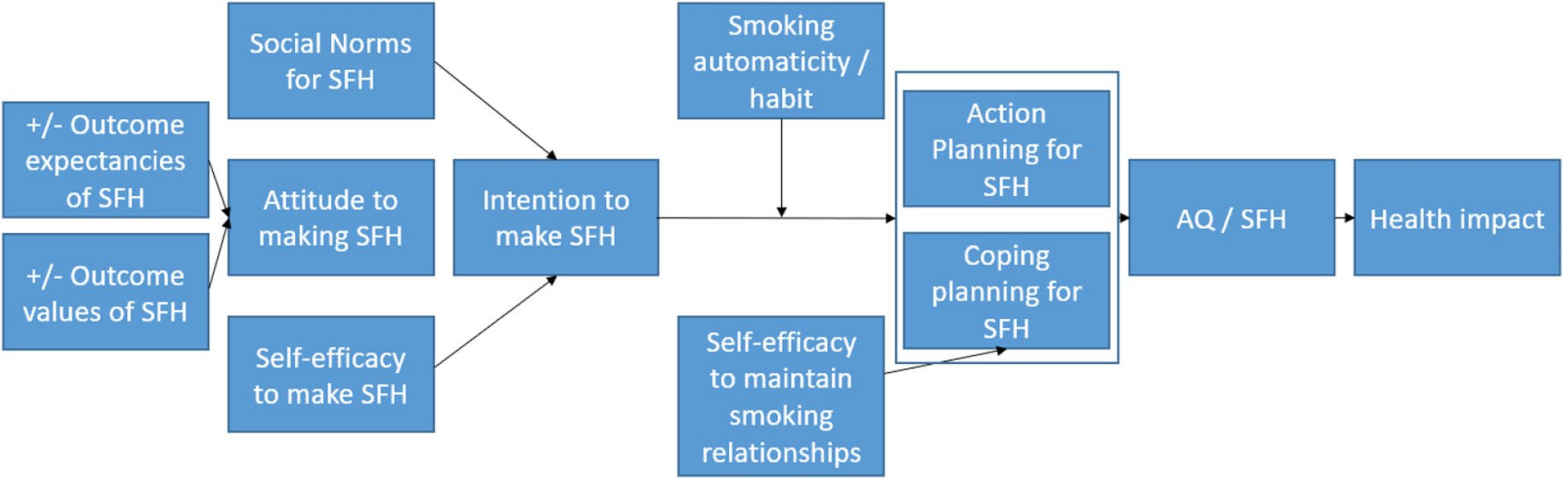
Intervention programme theory

Based on the evidence of the previous utility of the model for understanding and intervening on smoking behaviour [44, 45], we selected the Theory of Planned Behaviour, extended with action planning and coping planning as the starting basis for the programme theory to guide the development of the intervention content (see Fig. 1) targeting SHS. The constructs we sought to operationalise drawn from this model were Attitude, Social Norms, Intention formation, Self-efficacy, Action Planning, and Coping planning. Using the interview findings and the selected constructs from the programme theory, a Muslim colleague with knowledge of social cognitive constructs and the BCT taxonomy [41] supplied a list of ayahs that could support messages to promote change in these potentially modifiable constructs that were identified as being present within the interview. The programme theory constructs findings were then mapped to BCTs [41] that seemed likely to result in changes in those constructs based on study team expertise and subsequently result in change in air quality (AQ) and smoke-free home (SFH) status. The list of ayahs, the constructs targeted, the health messages, and the BCTs the health messages were mapped to is contained in Table 4.

**Table 4:** Initial and post-feedback ayahs, constructs, messages, and coded BCTs

Week	Constructs	Pre-feedback		BCT	Post feedback	
		Ayah	Message		Ayah	Message
1st	Attitude	Sura Al Baqara–219 (2:219) They ask you about drinking and gambling. Say, “There is great harm in both, though there is some benefit also for the people. But the harm of the sin thereof is far greater than their benefit”.	Though sometimes people think that smoking helps in some ways, the evidence that smoking and second-hand smoke cause harm in many ways is clear. Would Allah permit you something harmful? No! Tobacco is harmful, and hence it is not permissible to Allah. The sin of smoking causes you spiritual as well as physical harm.	5.1 Information about health consequences 5.2 Salience of consequences 5.6 Information about emotional consequences 5.3 Information about social and environmental consequences	Surah Al-Maaida-Ayah 4 (5:4) They ask you, [O Muhammad], what has been made lawful for them. Say, “Lawful for you are [all] good foods.”	[Unchanged]
2nd	Attitude	Sura An-Nisaa–59 (4:59) Believers! Obey Allah and obey the Messenger, and those from among you who are invested with authority	Allah has in His grace given us experts who he has been given authority to tell us the facts about what heals us and what harms us. The evidence from scientists tells us that second-hand smoke contains more than 7000 chemicals. Hundreds are toxic and about 70 can cause cancer. Second-hand smoke also causes numerous health problems in infants and children. Will you not listen to the facts? Will you not hear what your Imam says to you?	9.1. Credible source 5.1 Information about health consequences 5.2 Salience of consequences 5.6 Information about emotional consequences 5.3 Information about social and environmental consequences	[Unchanged]	[Unchanged]
3rd	Social norms	Sura Al-Ahzaab–58 (33:58) And those who harm believing men and believing women for [something] other than what they have earned have certainly born upon themselves a slander and manifest sin.	The evidence that second-hand smoke harms other is clear. It can result heart attacks, stroke, and lung cancer among innocent adults who are exposed to it. And children exposed to second-hand smoke are more prone to have chest infection, sneezing and coughing. Moreover, they have a 50% higher chance of having ear infection. Now do you really want to do that to your family members and your children? Similarly, Allah has said—causing harm to others is a manifest sin.	6.ϯ. Information about others Approval 5.1 Information about health consequences 5.2 Salience of consequences 5.6 Information about emotional consequences 5.3 Information about social and environmental consequences	[Unchanged]	The evidence that second-hand smoke harms other is clear. It can result heart attacks, stroke, and lung cancer among innocent adults who are exposed to it. And children exposed to second-hand smoke are more prone to have chest infection, sneezing, and coughing. Moreover, they have a 50% higher chance of having ear infection. Now do you really want to do that to your family members and your children?
4th	Intention formation (and prompt action planning)	Sura At-Takaathur—8 (102:8) Then, on that Day, you will be called to account for all the bounties you enjoyed.	These messages to you are part of Allah’s bounty to you. But you need to make a commitment to enjoy his bounty. This means committing to either quitting or smoking outside. If you are going to do this, you need to make a plan. For quitting smoking at home, commit that if you reach for a cigarette—then leave the house before you light it. And for planning to quit smoking completely, commit that if you feel like smoking, then pray 2 rakat salat instantly.	1.1. Goal-setting (behaviour) quit attempt 1.3. Goal-setting (outcome) smoke free home 1.4. Action planning 1.9. Commitment	[Unchanged]	[Unchanged]
5th	Self-efficacy (prompt action planning)	Sura Ar-Ra’d – 11 (13:11) The fact is that Allah does not change a people’s lot unless they themselves change their own characteristics	You can trust Allah to help you, but to receive that support, you must take a step by yourself in faith. Trust that Allah will give you everything you need. You can feel it difficult to quit smoking at home. But if YOU cannot make this simple change of behaviour for the sake of your family members, how can you expect Allah will help them in other ways? So, you need to make a plan that if you feel like smoking when you are at home—then leave the house before you light it.	3.1. Social support (unspecified) 1.4. Action planning 1.9. Commitment	[Unchanged]	[Unchanged]
6th	Coping planning	Sura Nooh—10–12 (71:10–12) I said to them: 'Ask forgiveness from your Lord; surely He is Most Forgiving. He will shower upon you torrents from heaven, and will provide you with wealth and children, and will bestow upon you gardens and rivers.	Allah knows you, Allah knows everything. He knows that you will need his forgiveness. Be quick to come to Him. Trust that He will be with you as you come back to the right path. So make a plan that if you lapse, then you will call on Allah for forgiveness and recommit yourself and rehearse your plans.	3.1. Social support (unspecified) 1.4. Action planning 1.9. Commitment	Surah Al-Maaida—Ayah 9 (5:9) Allah has promised those who believe and do righteous deeds [that] for them there is forgiveness and great reward.	[Unchanged]
7th	Attitude	Sura Al Maaida—90 (5:90) Believers! Intoxicants, games of chance, idolatrous sacrifices at altars, and divining arrows are all abominations, the handiwork of Satan. So turn wholly away from it that you may attain to true success.	Tobacco is toxic. Your body becomes reliant on nicotine. It doesn’t relieve stress. It only relieves withdrawal syndrome from your addiction. Tobacco is the handiwork of Satan. Do you want true success? Turn away wholly from tobacco.	5.1 Information about health consequences 5.2 Salience of consequences 5.6 Information about emotional consequences 5.3 Information about social and environmental consequences	[Unchanged]	[Unchanged]
8th	Attitude	Surah Al-Maaida–Ayah 100 (5:100) Say, “Not equal are the evil and the good, although the abundance of evil might impress you.” So, fear Allah, O you of understanding, that you may be successful.	A number of you may believe that smoking is good because it helps keep you warm or stops you getting fat, or manages your stress. But Allah, in his grace, has given us eye to see, ears to hear and a mind to enquire. What do the experts tell us? Experts tell us that it does nothing but harm you and those who are staying beside you when you are smoking. The only relief you feel getting after smoking is the relief from withdrawal syndrome which we mistakenly think as stress relief.	9.1. Credible source 5.1 Information about health consequences 5.2 Salience of consequences 5.6 Information about emotional consequences 5.3 Information about social and environmental consequences	[Unchanged]	[Unchanged]
9th	Social norms	Sura At-Baqara–195 (2:195) And do good; indeed, Allah loves the doers of good.	Globally 6 million people die every year from smoke. Those who smoke among us are directly causing harms to others unknowingly. So, we need to be aware and careful about that. We need to take away these messages to others. We need to make our families safe from this harm.	6.ϯ. Information about others’ approval 5.1 Information about health consequences 5.2 Salience of consequences 5.6 Information about emotional consequences 5.3 Information about social and environmental consequences	[Unchanged]	[Unchanged]
10th	Intention formation	Sura Al-Baqara:269 (2:269) He gives wisdom to whom He wills, and whoever has been given wisdom has certainly been given much good. And none will remember except those of understanding.	Allah has given you wisdom, but to remember it, you have to act on it. Only then you and others will be benefitted by it. If you are going to do something, you need to make a plan. For example, if you reach for a cigarette when you are at home—then leave the house before you light it. And for quitting smoking, you should plan like this—if you feel the urge to smoke, pray 2 rakat salat instantly.	1.1. Goal-setting (behaviour) quit attempt 1.3. Goal-setting (outcome) smoke-free home 1.4. Action planning 1.9. Commitment	Surah Ash-Shams—Ayah 7 to 10 (91:7–10) And [by] the soul and He who proportioned it. And inspired it [with discernment of] its wickedness and its righteousness, He has succeeded who purifies it, and he has failed who instils it [with corruption].	[Unchanged]
11th	Self-efficacy (prompt action planning)	Sura At-Talaaq-4 (65:4) And whoever fears Allah—He will make for him of his matter ease.	Those who smoke can find it difficult to quit smoking or they can find it hard to go outside home every time they want to smoke. But believe it, Allah will help you if you wish to listen to him. One can make simple plans to overcome such issues. Just commit to yourself and others (if you can) that whenever you feel the urge of smoking, go outside home to light it or pray 2 rakat salat instantly.	3.1. Social support (unspecified) 1.4. Action planning 1.9. Commitment	Surah At-Taghaabun—Ayah 16 (64:16) So, fear Allah as much as you are able and listen and obey and spend [in the way of Allah]; it is better for yourselves. And whoever is protected from the stinginess of his soul—it is those who will be the successful.	[Unchanged]
12th	Coping planning	Sura Luqman – 17 (31:17) Son, establish Prayer, enjoin all that is good and forbid all that is evil, and endure with patience whatever affliction befalls you. *29 Surely these have been emphatically enjoined.	Allah knows best about His creatures. He understands that we may do things that will harm us and others. That is why, he encouraged us to enjoy all that is good and forbid all that is evil and keep patience in times of affliction. We must remind ourselves these words of our creator again and again. We must try to make our habits safe for others. We must remember the possible harms of our behaviour to others like smoking at home and repetitively plan to keep us and our families safe from its harm.	3.1. Social support (unspecified) 1.4. Action planning 1.9. Commitment	Surah Al-Hajj—Ayah 77 (22:77) O you who have believed, bow and prostrate and worship your Lord and do good—that you may succeed	[Unchanged]

### Phase 3—User testing of candidate intervention content results

All participants understood the general meaning of the ayahs and the health messages as well as the links between the two. Small edits to the precise wording of some of the public health messages were made, to improve comprehension; for example, for the message linked to Ayah Sura At-Takaathur (see Table 4, ayah 4), the concept of “worldly pleasure” was unclear to some leading to a suggestion to reword this. No major changes were deemed necessary at this stage.

### Phase 4—Iterative intervention development workshops with Imams and khatibs Imams’ experience and views of delivering health promotion messages

There was a view among the Imams that they talk about health-related issues in the mosques only when directly relevant to religion, for example, addiction to smoking or alcohol or eating good foods; or when prompted by a current public health issue such as an outbreak of disease where they may advise on disease prevention strategies.

Addiction and smoking are sometimes discussed in mosques because it is destroying our children and adults, taking them away from Allah. There are young people who are always behaving badly to their parents. They are acting unaware of the consequences both in this world and the hereafter. (B07: Imam, khatib and Principal)

Allah has even told us to eat pleasant foods... Drugs, smoking, these are already Haram by Allah's law and moreover there are unpalatable, stinky food, which is why these are harmful for health. (A06: Imam)

A few days ago, city corporation people came to us and told us to talk on Chikungunya in Jumu’ah prayers. So, we did this. (B03: Imam and khatib)

The exception was during Ramadan when there is more emphasis on changing people’s “bad” behaviours and helping them to focus more on praying to Allah.

They were generally motivated to deliver health messages in mosques and familiar with including messages during Khutbah in Jumu’ah prayer about behaviours that harm people both physically and spiritually. Educating men about the risks of smoking and SHS was seen as a good idea, particularly as people rarely learn about SHS, so the intervention was considered to represent an opportunity, with the input of international researchers seen as an asset. Additionally, this perceived scientific foundation of the intervention was seen as important as Imams did not consider themselves experts on public health, rather their expertise was in spiritual matters.

Actually, you have to pray to Allah from Dunya (this world). After death, there is no chance for earning good deeds. So, for earning good deeds, the first condition is Haya (life). Abstaining from addiction what Allah prohibited and what the prophet (PBUH) did and encouraged us to do, if we follow those, the Hayat will increase. (A01: Imam)

If we can tell them about some medical facts on smoking along with religious messages on it, they will be more aware of it. (B04: Imam)

We have both indirect and direct smoking here which is very bad. People do not hear much about second-hand smoking from anyone I guess. (B02: Iman and Teacher)

So, if we get a booklet or guideline including information on medical science, and if the messages are included by studying Quran and Hadith, then these will be more acceptable. People will understand that not only Imams know about Quran and Hadiths but also are knowledgeable of other fields. (A02: Iman and khatib)

They were also happy to deliver messages about planning, attempting, and failing to change behaviours, observing that people are used to this, and Islam teaches them how to face such situations, with Imams seen as a trusted source of support.

I think this is a great opportunity for Imams and common people because thousands of people can be reached with these messages and thus, Imams can make more people aware. (A05: Imam and khatib)

Jumu’ah prayers on Fridays was seen as the most appropriate time to deliver the messages, as this is when there are large numbers of people in the mosque, and they have time to elaborate on the meaning. There was a view among some that it would be important to deliver a message one week, discuss it the next week, and then return to it several weeks later as a reminder.

#### Feedback on ayahs and health messages

Imams were keen to undertake a careful check of the selected ayahs and proposed links with health messages. Some wanted more time outside of the workshop to do this work, whilst others advised that alims (Islamic scholars) should review the final list of ayahs and associated health messages.

There was agreement that the same ayahs and linked public health messages were appropriate for all mosques. The Imams’ suggestions for the 12 ayahs (listed in Table 4) are summarised below. The consensus across both workshops was that ayahs 3, 5, 7, 9, and 12 were appropriate and that ayah 4 was not suitably linked to the public message, although no one had an idea for a replacement. For the others, suggestions for alternatives were offered. These were usually to avoid misinterpretation or strengthen the take-home message. For two ayahs, changes were proposed to correct the meaning in the context of Islamic scripture.

Ayahs 1 and 10 were considered by some Imams to be open to misinterpretation. For Ayah 1, there was some concern that people might think that smoking is beneficial. Ayah 10 was seen as confusing about the type of knowledge being referred to; it should be understood to be knowledge of religion not knowledge of the harms of SFH. For ayahs 6 and 12, some Imams wanted to strengthen the message about the forgiveness of Allah. Alternatives for ayah 8 were offered to further encourage people to change their smoking and second-hand smoke behaviours by emphasising the importance of following the life and guidance of the prophet.

The two ayahs that were questioned in terms of religious accuracy were 2 and 11. For ayah 2, precision was needed that it is the Imam (not the scientist) who has authority to advise on what harms and heals to be consistent with the laws of Sariah. For ayah 11, the selected ayah was referring to divorce hence inappropriate.

As a result of the workshops, half the Ayahs were replaced with different Ayahs that better conveyed the messages or were more closely related to the public health messages targeted to be delivered. Ayahs 1, 6, 8, 10, 11, and 12 were changed. Ayahs 1, 8, 10, 11, and 12 were replaced with Ayahs suggested by the scholars of the Islamic Foundation, Bangladesh, and Ayah 6 was replaced with another Ayah chosen by ARK researchers (see Table columns 6 & 7).

#### Format of the intervention content

**Fig. 2:**
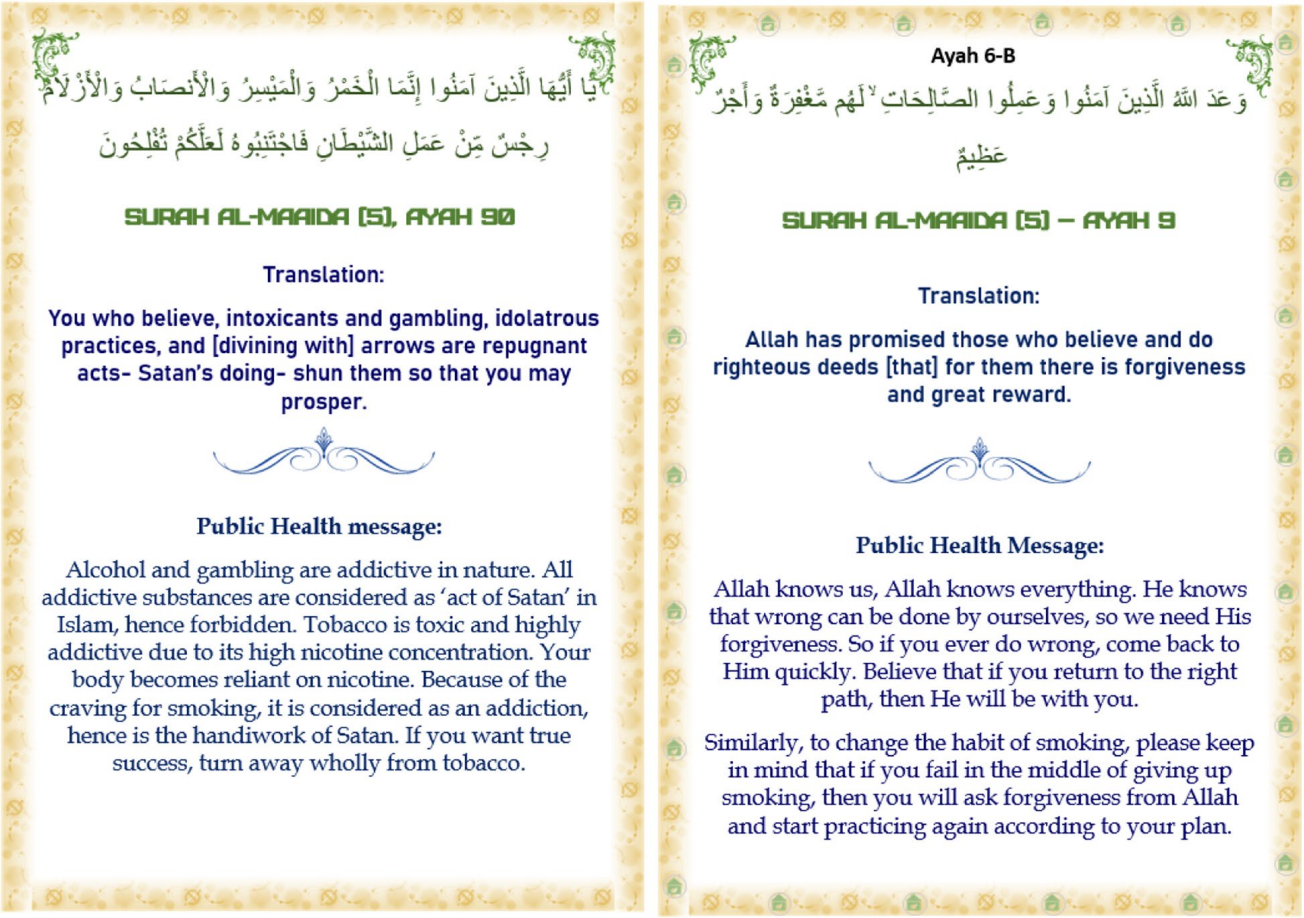
Examples of pages of the intervention booklet (translated into English)

The final version of the intervention was formatted as a booklet for Imams that contained the Arabic ayah, a translation into Bangla, and the related health message (see Fig. 2 for examples translated into English).

The intervention booklet finally contained 12 ayah and related health messages in total (see Table 1 columns 6 & 7). Training on delivery of the intervention was provided over a half-day and was supported by a training manual. Training materials are available at [https://www.york.ac.uk/healthsciences/research/public-health/projects/mclass11/#tab-3]. Imams or khatibs in the mosques that were randomised to deliver the SFH intervention received copies of the intervention booklet to distribute to their congregation members after Friday Jumu’ah prayers or in study circles. Intervention delivery started immediately after training and continued for 12 weeks. Full details of the trial procedures have been previously published [9].

## Discussion

The intervention development process reported here primarily took an evidence and theory-based approach [34], based on the MRC Framework [46, 47], in common with multiple approaches to intervention development [48]. Additionally, we took a partnership approach and engaged with stakeholder groups to both generate ideas about components and features of the intervention [49] and make decisions about the content, format, and delivery of the intervention [48]. As such, this was a combination approach to intervention development [34].

### Summary of this approach

In accordance with MRC guidance [46], considerable resources were invested to develop an intervention with a conceivable intervention effect on SFHs. This process benefitted from intervention development that had previously been undertaken as part of the UK MCLASS trial [1, 24, 26], as well as intervention development work that preceded this [29]. The four phases undertaken were resource-consuming. However, each phase either directly or indirectly supported the creation or adaption of intervention content, with interviews exploring barriers and facilitators of SFH with adults, subsequent identification of an intervention programme theory and population of initial content with Quranic scripture, user testing of candidate intervention content with adults that resulted in minor changes to aid understanding, and iterative intervention development workshops with Imams and khatibs that resulted in major changes to the content to better reflect Islamic scholarship. The paucity of evidence as to effective SFH interventions [5, 6], and the previously highlighted concerns about intervention content [24], provided the impetus to appropriately support engagement with stakeholders to understand the religious and socio-cultural sensitivities of promoting SFH in a mosque setting [30, 50]. This approach reflects calls to conceptualise stakeholder involvement as an ongoing, iterative process [51, 52], and represents the efforts to develop shared terminology, successful prioritisation of early and consistent engagement, and recognition of stakeholders’ contributions [53].

### Limitations

This intervention has subsequently been trialled [9] and found not to be effective in reducing household SHS exposure compared with usual services. However, further process evaluation and analysis of secondary outcomes [25] is planned that will explore effects on hypothesised intervention casual pathways and intervention fidelity [54].

We benefited from generous support from colleagues with deep knowledge of ayahs, social cognition models, and/or the behaviour change technique taxonomy [41]. Additionally, access to the Quranic Search Tool [42] provided a starting point for engagement with faith leaders that would have been difficult to replicate without significant external support. The ease with which these resources can be replicated is not obvious but speak to the necessity to properly resource intervention development and/or adaptation activities in culturally sensitive settings [53].

This work predates a landmark series of studies [55–57] that triangulated evidence for links between social cognitive constructs and BCTs [41]. Whilst prior to the availability of the Theory and Technique Tool that resulted from these studies, it was typical as part of an intervention development process to make use of study team expertise to map social cognitive constructs identified through qualitative or quantitative inquiry to BCTs, and this is a less robust method than the evidence synthesis and expert consensus approach that provided the data that is now available to support the mapping of such links. As such, the BCT mapping upon which we based our selection of ayahs may be less than optimal.

## Conclusion

This religious community-based intervention to reduce SHS exposure at home and improve lung health among non-smokers in Bangladesh is the result of an iterative and collaborative 4-stage process. It makes use of behaviour change theory to support faith-community contributions to the production of culturally sensitive intervention content suitable for a mosque-based setting. Whilst further process evaluation is necessary to understand its failure to affect SHS [9], this novel combination of intervention development framework components demonstrates a flexible approach that could provide insights for intervention development in related culturally sensitive contexts that could support health behaviour change.

## Supplementary Information

**Additional file 1.** **Appendix 1.**

**Additional file 2.**

## Data Availability

De-identified participant data will be made available from the point of and up to 5 years after the acceptance for publication. These data can be requested from the Principal Investigator (Prof Kamran Siddiqi; kamran.siddiqi@york.ac.uk) and will be shared after the provision of a methodologically sound proposal, and only under a data-sharing agreement that provides for commitment to using the data only for research purposes and not to identify any individual participant, securing the data using appropriate computer technology, and destroying or returning the data after analyses are completed. The proposals will be assessed and approved by members of the Programme Management Group. The intervention manual and indoor-air-quality feedback leaflet are available on the study webpage: https://www.york.ac.uk/healthsciences/research/public-health/projects/mclass11/#tab-3.
